# Continuous quantitative mapping of multi-organ T1 relaxation times with ShMOLLI to assess dose response in respiratory challenges at 3T

**DOI:** 10.1186/1532-429X-15-S1-W9

**Published:** 2013-01-30

**Authors:** SK Piechnik, V Ferreira, S Neubauer, MD Robson

**Affiliations:** 1 Cardiovascular Medicine, University of Oxford, Oxford, UK

## Background

The performance of T1 mapping as a stable biomarker depends on its sensitivity to pathological changes and relative insensitivity to normal physiological variations or common clinical interventions, such as oxygen administration. We address the impact of breathing air supplemented with oxygen (O2) and/or carbon dioxide (CO2) on T1 values in healthy myocardium and selected extra-cardiac tissues.

## Methods

**Figure 1:**
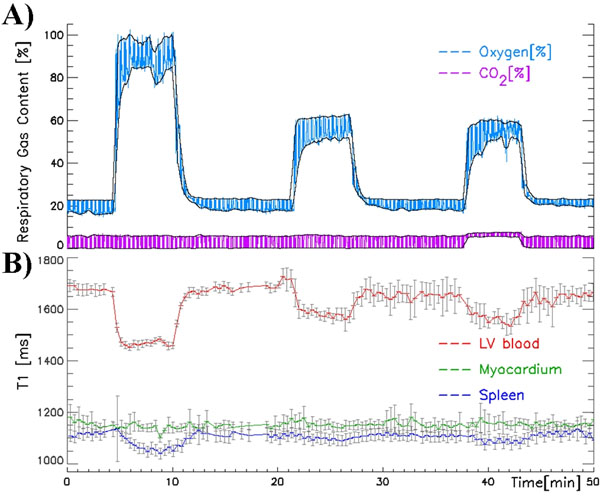
A) An example of respiratory monitoring traces with in- and end-tidal outlines marked in black during 100% O2, 60% O2 and combined 60% O2+5% CO2 challenges. B) Corresponding T1 trends in left ventricular blood, myocardium and spleen.

6 normal control volunteers (age 35±4 years, 2 females) underwent serial T1-mapping using the Shortened Modified Look Locker Inversion Recovery (ShMOLLI, Piechnik et al. JCMR 2010, 12:69) at 3T (Siemens Trio). Subjects breathed through a mouthpiece to allow monitoring of O2 and CO2 content (Datex Normocap 200) during three 15-minute respiratory challenges as follows: 4 minutes of normal air, 5 minutes of supplemented target mixture with O2 (21-100%) and CO2 (0-5%), and 6 minutes of normal air (Fig. 1A). T1-mapping was performed back-to-back at end-expiration under automated breathing instructions. Regions of interest (ROIs) were drawn on T1 maps within the myocardium, left ventricular (LV) and right ventricular (RV) blood pools, liver parenchyma excluding major vessels, and the spleen. ROIs were followed semi-automatically for motion between breath-holds. The relationship between changes in T1 in each organ and the end-tidal (Et) gas content was assessed using general linear modelling.

## Results

**Figure 2:**
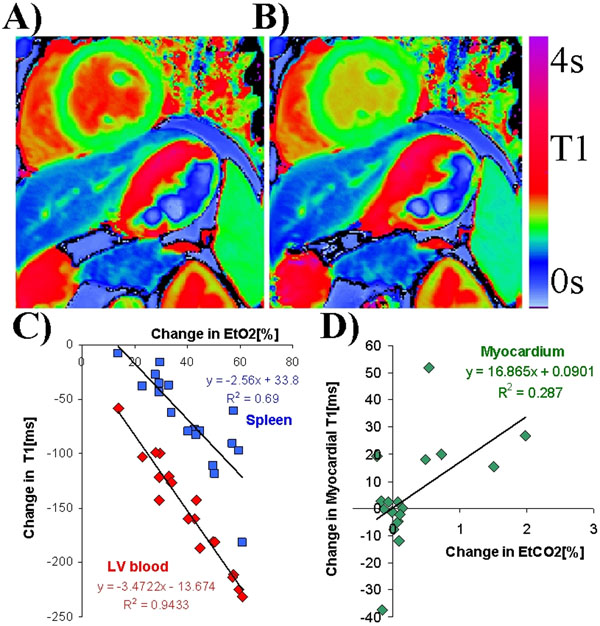
A) Colour T1 map at baseline. B) Colour T1 map at the end of 100% O2 challenge.C) Dependence of T1 in left ventricular blood and spleen on expired O2 content variation. D) Relationship between change in myocardial T1 and change in EtCO2.

Each 1% increase in O2 and CO2 resulted in a 0.76±0.2% and 0.3±0.04% change in end-tidal content, respectively. T1 maps were acquired typically every 23 seconds (<25s in 95% of the 701 measurements). Following O2 administration, LV blood and spleen demonstrated clear and visible lowering in T1 trendlines (Fig. 1B) and directly on colour T1 maps (Fig. 2AB) and. The relationships showed significant negative linear regression slopes of -3.5±0.2ms/%EtO2 (R2=0.94) and -2.6±0.4ms/%EtO2 (R2=0.69), respectively (Fig. 1C). There was no relationship between T1 in LV blood or spleen with CO2. T1 in healthy myocardium was not sensitive to changes in O2. Myocardial T1 increased slightly with CO2 administration (+17±7ms/%EtCO2, R2=0.29), corresponding to only a 3% deviation from baseline T1 within the limits of a typical 5% CO2 vasodilatory challenge (Fig. 1D). T1 in RV blood and liver did not correlate with O2 or CO2 content.

## Conclusions

ShMOLLI T1-maps can be acquired at a frequency of up to 3 per minute with excellent measurement consistency over extended periods of time, with potential use in the monitoring of physiological effects of respiratory challenges. T1 directly detects hyperoxygenation in arterial blood and its propagation downstream to some major organs such as the spleen. There was little evidence of major variation of T1 with O2 or CO2 in the other organs studied. Specifically, in comparison to normal T1 variability, myocardial T1 is relatively stable under feasible hyperoxia and hypercapnia.

## Funding

National Institute for Health Research (NIHR) Oxford Biomedical Research Centre.

